# The diversity of gendered adaptation strategies to climate change of Indian farmers: A feminist intersectional approach

**DOI:** 10.1007/s13280-016-0833-2

**Published:** 2016-11-22

**Authors:** Federica Ravera, Berta Martín-López, Unai Pascual, Adam Drucker

**Affiliations:** 1 ICAAM - Instituto de Ciências Agrárias e Ambientais Mediterrânicas, LDSP - Landscape Dynamics and Social Process Research Group, Universidade de Évora, Pólo da Mitra, Ap. 94, 7002-554 Évora, Portugal; 2 CREAF, Cerdanyola del Vallès, 08193 Catalonia, Spain; 3 Faculty of Sustainability, Institute of Ethics and Transdisciplinary Sustainability Research, Leuphana University, Scharnhorststr. 1, 21335 Lüneburg, Germany; 4 Basque Centre for Climate Change (BC3), Edificio Sede Nº 1, Planta 1ª, Parque Científico de UPV/EHU, Barrio Sarriena, 48940 Leioa, Spain; 5 IKERBASQUE, Basque Foundation for Science, Maria Diaz de Haro 3, 6 floor, 48013 Bilbao, Spain; 6 Department of Land Economy, University of Cambridge, 19 Silver St., Cambridge, CB3 9EP UK; 7 Bioversity International, Via dei Tre Denari 472/a, 00057 Maccarese, Rome, Italy

**Keywords:** Adaptation, Himalayan region, Indian Gangetic mid-plains region, Intersectionality

## Abstract

This paper examines climate change adaptation and gender issues through an application of a feminist intersectional approach. This approach permits the identification of diverse adaptation responses arising from the existence of multiple and fragmented dimensions of identity (including gender) that intersect with power relations to shape situation-specific interactions between farmers and ecosystems. Based on results from contrasting research cases in Bihar and Uttarakhand, India, this paper demonstrates, inter alia, that there are geographically determined gendered preferences and adoption strategies regarding adaptation options and that these are influenced by the socio-ecological context and institutional dynamics. Intersecting identities, such as caste, wealth, age and gender, influence decisions and reveal power dynamics and negotiation within the household and the community, as well as barriers to adaptation among groups. Overall, the findings suggest that a feminist intersectional approach does appear to be useful and worth further exploration in the context of climate change adaptation. In particular, future research could benefit from more emphasis on a nuanced analysis of the intra-gender differences that shape adaptive capacity to climate change.

**Electronic supplementary material:**

The online version of this article (doi:10.1007/s13280-016-0833-2) contains supplementary material, which is available to authorized users.

## Introduction

International initiatives, particularly the United Nations Framework Convention on Climate Change (UNFCCC) and the Sustainable Development Goals, recognize that understanding the vulnerability and adaptation responses of local communities to climate change is critical to promote mechanisms for climate change-related planning. In the context of climate change, adaptation refers to adjustments in natural or human systems in response to actual or expected climatic stimuli or their effects, which moderates harm or exploits beneficial opportunities (IPCC 2014). It involves adjustments in lifestyle, behaviours and socio-economic structures (i.e. livelihood security-based responses) as well as in land use and management of biodiversity and ecological processes (i.e. ecosystem-based responses) (Smit et al. 2000; Ojea 2015; Vignola et al. 2015). The social and institutional dimensions of vulnerability and adaptation to climate change have been increasingly addressed by scholars (Smit and Wandel 2006; McLaughlin and Dietz 2008), specifically opening questions related to understanding matters of social differentiation, equity and power (Füssel and Klein 2006). However, the multiple determinants that shape differentiated context-specific vulnerabilities and, particularly, adaptive capacity responses are still largely unexplored (Terry 2009; Carr and Thompson 2014; Kaijser and Kronsell 2014). Specifically, scholars recognize that such determinants are gendered and mediated by social, cultural, institutional and economic structures and processes (Morton 2007; Kaijser and Kronsell 2014; Sugden et al. 2014). In this sense, a significant body of literature on gender and climate change shows that women and men perceive and experience climate change differently, and usually women are more vulnerable due to their dependence on natural resources and structural inequity in their access and control of such resources (Dankelman 2010; FAO 2011; Nellemann et al. 2011; WEDO-IUCN 2014). However, recent critiques from feminist political ecology perspectives question the validity of the conventional binary male–female view of gender in climate change studies as they can lack sufficient consideration of power relations determined by the social context (Demetriades and Esplen 2008; Rodenberg 2009; Arora-Jonsson 2011; Resurreccion 2011; Carr and Thompson 2014). Firstly, presenting women as a passive victim of change may misinterpret the causes of vulnerability and obscure the role of women as proactive agents of adaptation (Mitchell 2007; Dankelman 2010). Secondly, climate change debates related to gender are shifting from a simple view of women as a homogenous group toward a more complex view of identities within gender categories (Tschakert 2012). Indeed, multiple social, economic, and cultural characteristics interact with gender in influencing power inequities and explaining how and why people face and manage climate change in different ways (Carr 2008). Thirdly, several authors recognize that climate change occurs together with other concomitant direct and indirect drivers, which together affect local livelihoods, gender relations and management decisions (O’Brien et al. 2004; Nielsen and Reenberg 2010; Sugden et al. 2014).

In this context, it is useful to introduce intersectionality—a feminist sociological concept first introduced by Crenshaw (1991)—which leads us to critically understand the nature of differentiated vulnerability and adaptation due to the interactions among multiple dimensions of social and power relations (McCall 2005; Davis 2008). Recently, a growing body of research from critical feminist political ecology has raised awareness about the importance of intersectionality on adaptation to climate change in agrarian settings (Carr and Thompson 2014; Kaijser and Kronsell 2014; Sultana 2014; Ogra and Badola 2015). In the Asian context, for instance, several empirical studies clearly confirmed the interplay of multiple identities, such as caste, economic class and gender, shaping differentiated vulnerability to risks and disasters (Ahmed and Fajber 2009; Ray-Bennett 2009; Onta and Resurreccion 2011). Other studies found that social class, household head gender, age and stage of life may determine women’s ability to respond to water scarcity (Huynh and Resurrección 2014). Similar interactions have been identified in Africa, where the joint effects of gender, access to education, land and credit are analysed as local determinants of the capacity to adapt to decreasing precipitation (Below et al. 2012; Fosu-Mensah et al. 2012). The intersectionality framework, thus, has potential application as an analytical tool for understanding how interactions among multiple social dimensions of power can determine the development of adaptation strategies to climate and other concomitant drivers of change (Adger and Kelly 1999; Nightingale 2011; Marino and Ribot 2012). However, methodological discussions on how to implement such a framework are open and empirical applications remain to be undertaken (Davis 2008; Lykke 2010; Kaijser and Kronsell 2014).

By adopting an intersectional analysis approach, this paper aims to investigate the role of gender and its interaction with cultural, social and economic factors in determining the adaptive responses to climate and other multiple drivers of change in two empirical cases in India, located in two regions extremely vulnerable to climate change. We specifically aim to answer three questions: (i) How do the responses to climate change and other concomitant agrarian pressures manifest themselves in different biophysical and socio-economic regions? (ii) Are local vulnerability and adaptation strategies gender-differentiated in their perception and adoption? (iii) Are there other cultural, social and economic factors interacting within the different contexts with gender, contributing to shape adaptation responses?

## Case studies

**Fig. 1:**
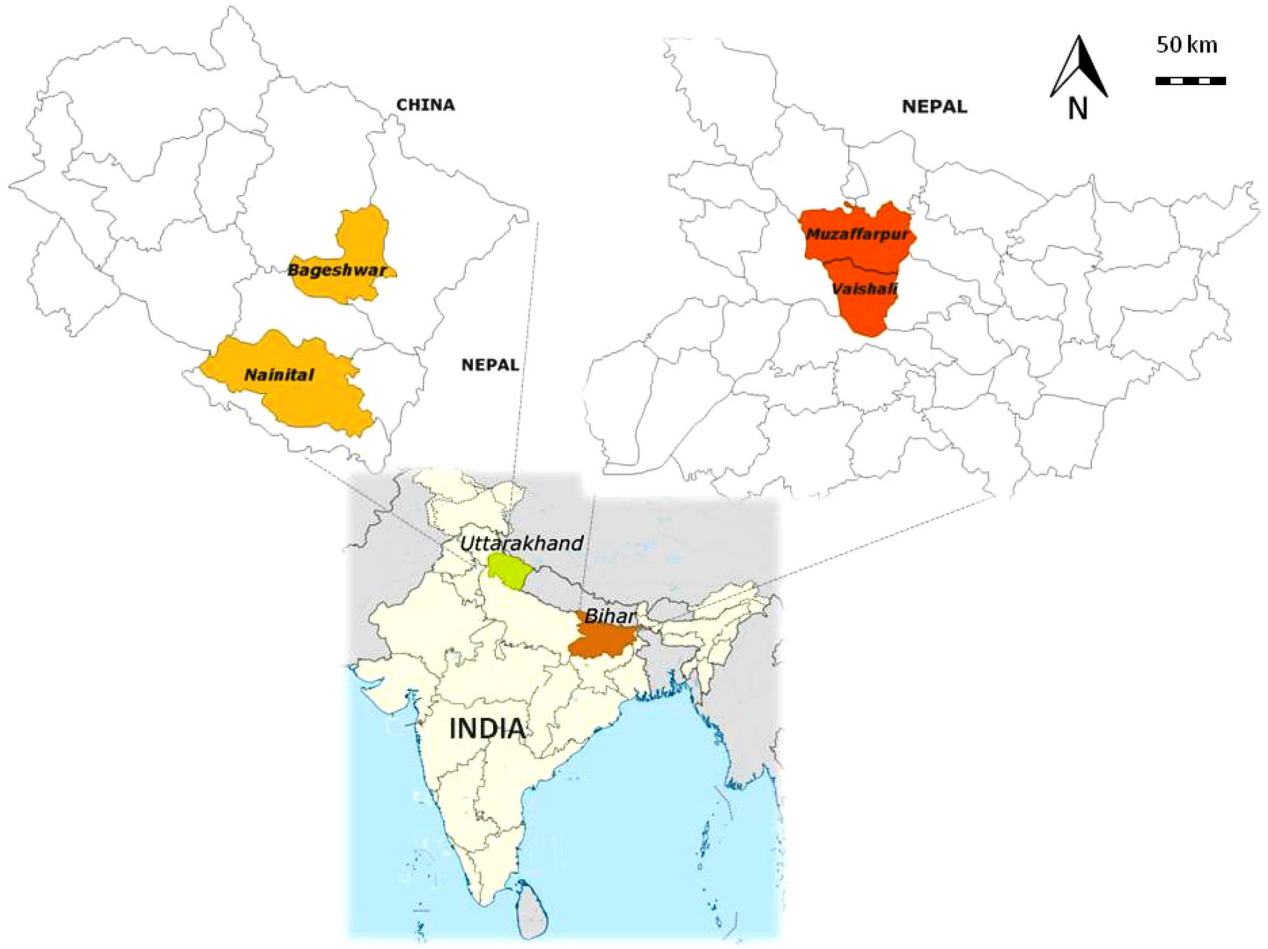
Location of the two case studies in India

The first case study was located in the Kumaon region of the Himalayan State of Uttarakhand, representing a mid-high hills agro-ecological zone (ca. 1200–1600 m above sea level). The second case study was located in an agro-ecological zone characterized by plains in the State of Bihar, in the mid Indian Gangetic region (ca. 30–50 m a.s.l) (Fig. 1).

### Mid-high hills Kumaon region of Uttarakhand

In the Kumaon region, farming is largely practised on terraced rainfed upland fields and lowland fields irrigated with traditional channels. In this region, the study was conducted in two sites, in the districts of Nainital and Bageshwar. According to our sample (see Table S1 in Supplementary material 1), most of farmers (72 % in Nainital and 85 % in Bageshwar) are marginal farmers with less than 0.4 ha of agricultural land. In Nainital, a small percentage of farmers (6.5 %) own 33 % of the land. Two main cropping systems coexist: (i) in the lowlands (prevalent in the Bageshwar site), farmers alternate irrigated rice (*kharif* season, from June to October) with wheat and barley (*rabi* season, from October to April); (ii) in the upland fields (prevalent in the Nainital site), farmers cultivate dry rice, millets (i.e. finger millet, pearl millet, foxtail millet, barnyard millet) and pulses (i.e. *horsegram*, *black soybean*, *kidney beans*, *black gram*, *pigeon pea*, *soy bean*, *lentils*, *mung bean*, *rice bean*, *simi*, *pea*) in the *kharif* season, in rotation with wheat and barley in the *rabi* season. Farmers in the two sites grow multiple crops through polyculture, including intercropping, crop rotation and the use of locally adapted and traditional varieties (i.e. landraces).

The State of Uttarakhand has relatively favourable indicators relating to women’s educational and health status compared to other States in India (Nautiyal 2003). In both sites, women are organized in informal networks to undertake agricultural activities such as soil fertility management and crop harvesting. Women and men adhere to culturally established and differentiated gender roles, including with regard to the division of labour. Such differentiation is associated with the traditional *pahari* (i.e. mountain) identity of the Kumaoni people (e.g. Pokhriyal 1994; Badola and Hussain 2003; Mehta 2008; Ogra 2008), which place women at the centre of the agricultural system, while men are expected to participate in the cash economy mainly related to off-farm income generation. Furthermore, in this region, Kumaoni people are an ethnic group with a traditional caste system^1^ that is still prevalent.

The Himalayan region has been also recognized as particularly affected by climate change (Shiva and Bhatt 2009; IPCC 2013). In Uttarakhand, climate change results in more intense and longer periods of drought, with decreasing snow events and late monsoons underpinning the lack of water resources in spring (Government of Uttarakhand 2014). These climatic changes act in tandem with other socio-cultural changes in demography, local economies (Jain 2010), agricultural practices and technology, food habits (Bisht et al. 2006; Nautiyal et al. 2008) and environmental change (e.g. deforestation, natural resources degradation and the introduction of invasive species) that may be of particular concern in the context of the increased vulnerability of women (Nellemann et al. 2011). In 2012, the Uttarakhand Action Plan for Climate change (UAPCC) recognized the increased feminization of agriculture and consequent vulnerability of women in the context of climate change, especially those small landholders that belong to the Dalit (i.e. the lowest) caste, and implemented a specific Women and Child Welfare Department.

### The Indian Gangetic mid-plains region of Bihar

The Ganges River Basin includes one of the most populated and agriculture-dependent regions of India. In the state of Bihar, agriculture is mainly practised on the plains. Two sites (a drought-prone one and a flood-prone one) were selected as being representative of the main cropping systems in the region (i.e. a rice/wheat cropping system in *kharif*/*rabi* seasons). In the first site, in Vaishali district, vegetables are also cultivated as cash crops, while in the second site, in Muzaffarpur district, tobacco cultivation for cash prevails. Intercropping and rotation are common in both sites. In Bihar, the agricultural cycle includes a third season, the *jaid* (i.e. March–June), when pulses are cultivated. In both sites, agriculture is fairly intensive, with access to irrigation facilities, mechanized ploughing and hybrid crop varieties.

In terms of socio-economic structure, a percentage of farmers are either landless (10.3 % in Vaishali and 6.7 % in Muzzafarpur) or marginal farmers with less than 0.5 ha of agricultural land (38.2 % in Vaishali and 27.1 % in Muzzafarpur), while the majority are of medium size. In Vaishali, farmers with large extension of land (more than 2 ha and up to 15 ha in our study area) represent only 4 % of the sample and own 8 % of land, while in Muzaffarpur this category occupies more than 55 % of the total land (Table S1 in Supplementary material 1). Due to this unequal access to land, the *Batai* farming system, sharecropping through informal arrangements, has been historically practised in Bihar. Despite the deeply exploitative nature of this system (i.e. most tenants have to pay input costs), it is the main mechanism adopted to guarantee access to land for landless and very small farmers.

Historically, in this region the social structures and taboos from classes and caste^2^ have resulted in restrictions applying to both Muslim and Hindu women. Such restrictions determine land inheritance laws, which exclude women (Agarwal 2002), and task involvement in agriculture (e.g. women from tenant families are mainly net labour buyers in the area). Today, women’s roles and mobility outside the *tola* (i.e. traditional area of the village occupied by a caste) are changing (Datta and Rustagi 2012). As a result of male out-migration in poor and low caste households of Bihar, women’s involvement in the agricultural workforce is even greater as women have become the de facto head of the households (Rao 2006; Binswanger-Mkhizer and D’Souza 2012).

In the Indian Gangetic plains, the combined pressures of population growth and climate change are likely to have significant impacts on agriculture and social dynamics in the years to come (Ministry of Home Affairs 2011). In the specific context of Bihar, climate change is expected to impact due to heat stress (IPCC 2014), erratic monsoon events, with repetitive drought and unexpected floods (Sehgal et al. 2013; Kishore et al. 2014). Additional environmental hazards can result in the degradation of the quality and quantity of water, as well as being related to soil erosion (Mall et al. 2006; Sinha 2011). Since 2009, Bihar has experimented four major droughts. In response, numerous external interventions involving women have sought to provide training and external support for implementing smart agriculture practices along with technological practices to mitigate the effects of climate change (Mehar et al. 2016). For instance, at the Vaishali site, a formal women’s organization was created as part of the CGIAR Research Program on Climate Change, Agriculture and Food Security (CCAFS). In Muzzafarpur, researchers from Rajendra Agricultural University, among others, have been working with local farmers on agro-biodiversity management programmes. However, the Bihar Action Plan for Climate change (Government of Bihar 2014) makes little mention of the statutory and customary rules that restrict women’s access to land, information and services. These constitute barriers to engage in environmental restoration and management programmes and are a cause of major vulnerability to climate change hazards.

## Materials and methods

### Data collection

Data collection was conducted between July and December 2012 in the two districts selected in each of the two case studies.^3^ A total of seven villages in Uttarakhand and six villages in Bihar were selected.

**Fig. 2:**
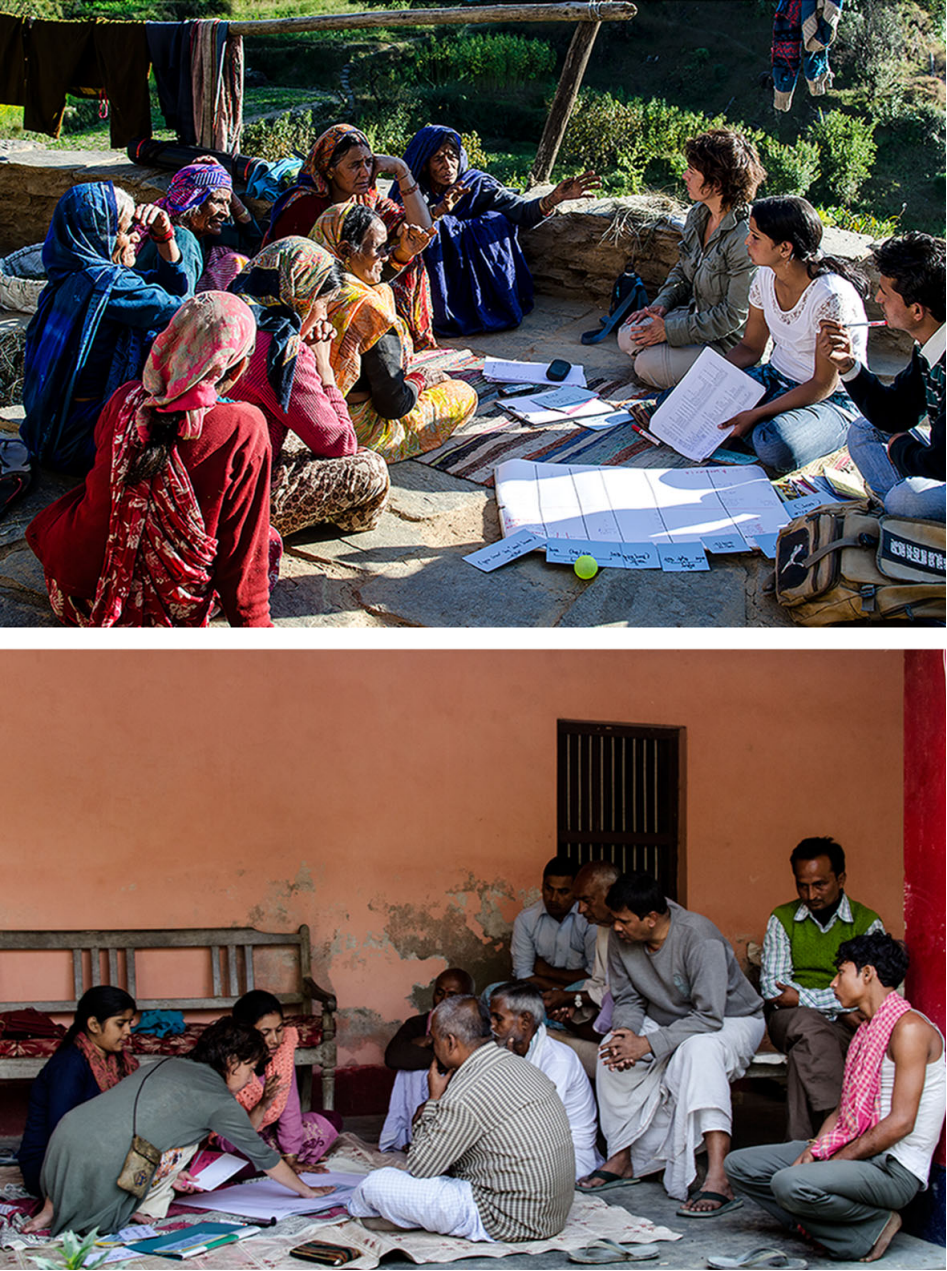
Focus group discussions with women in Uttarakhand (*top*) and with men in Bihar (*bottom*) (Photo: David Tarrasón)

Qualitative information was collected in order to explore the differentiated perceptions of men and women about climate change as well as other drivers of change, along with the strategies they rank as the most relevant to counteract the effect of these drivers. Along with secondary data, information was collected, in the first instance, through in-depth interviews with key informants, local NGOs, local scientists and governmental representatives. Field surveys were conducted with the assistance of two translators: a woman to talk mainly with women and a man to talk mainly with men. Focus group discussions (11 in Uttarakhand and 14 in Bihar) (Fig. 2) were realized with the assistance of two translators (a woman and a man), as female and male focus groups were organized separately. In Bihar, separate focus groups were organized for upper and lower castes in one village and for farmers who were participating or not involved in external projects and programmes in a second village. Participants first discussed and prioritized the main drivers of change (i.e. climate change and other environmental change; socio-economic and political change; cultural change) and related impacts. Then, they listed and ranked the adaptation strategies considered as relevant for reducing the vulnerability of local livelihoods or for managing and mitigating the impacts of these drivers. The strategies were ranked in each focus group from 5 (very important) to 0 (not important). This information on the ranked adaptation strategies was then used to design the larger household survey.

The household survey was designed to disentangle the key factors that determine the differentiated application of adaptation strategies in the two research zones. A total of 136 and 178 semi-structured interviews were conducted in Uttarakhand and Bihar, respectively. In each village, we used a stratified random sampling design. Stratification was based on the diversity of castes and/or agro-ecological conditions within the sampled villages. Although we attempted to have a stratified random sample in order to avoid gender bias, interviewing women in Bihar was challenging due to their general seclusion, being under-represented in our sample (59 % men and 41 % women). The final survey contains four sections: (i) general information regarding the respondent, such as gender, caste, education and age; (ii) other demographic and socio-economic information relating to the household; (iii) gender participation in decision-making and gender division of tasks and responsibilities among household members in agricultural activities such as seed nursery, ploughing, sowing and transplanting, harvesting, storing, post-harvesting and marketing and (iv) identification of drivers of change, impacts on local livelihoods and strategies adopted by the household to cope with and adapt to such drivers.

### Data analysis

A mixed qualitative–quantitative data analysis was performed in two stages. In the first stage, the information collected through focus groups was transcribed and systematized to identify and rank the relative importance of adaptation strategies to climate change and other drivers of change, as well as their impacts. This information was prioritized in each zone according to gender differences. Drawing on Vignola et al. (2015), each of the strategies was classified into ‘Ecosystem-based adaptation strategies’ and ‘Social, cultural and economic-based adaptation strategies’. Ecosystem-based adaptation strategies were classified into two dimensions: ‘conservation, restoration and sustainable management of (agro)biodiversity’ and ‘conservation, restoration and sustainable management of ecological functions and processes’. Similarly ‘social, cultural and economic-based adaptation strategies’ included the dimensions of ‘livelihood security’ and ‘societal ties and knowledge management’. A third category of adaptation strategies associated with technology was also considered: ‘technological strategies’. In addition, strategies were classified into ‘proactive strategies’ (i.e. planned or traditional actions which take place in anticipation of climate change or other stressors) and ‘reactive coping mechanisms’ (i.e. actions which occur after the fact with a view to managing impacts *ex post*) (Morton 2007; Ravera et al. 2011).

The second stage entailed quantitative data analysis. Canonical correspondence analysis (CCA) was applied as a multivariate ordination technique to explain the variation of adaptation strategies (dependent variables) according to a group of explanatory variables. Three main gender-sensitive variables were included: (1) gender of respondent, (2) the degree to which decision-making in the household is gendered and (3) the degree of gender involvement in agricultural tasks. Besides these gender-sensitive variables, we included the following additional variables in order to apply the intersectionality framework: socio-demographic and cultural characteristics of households and respondents, i.e. size of household, age, caste, education, social capital, household’s access to multiple assets (i.e. a proxy of wealth), farm location and level of perception of drivers of change (see Supplementary material 1). By applying a CCA, we were able to analyse the joint interaction between gender-sensitive and other variables on the decision of applying different adaptation strategies. The significance of the association between adaptation strategies and the explanatory variables was evaluated using a Monte Carlo permutation test (500 permutations). CCA analyses were performed separately for Uttarakhand and Bihar.

## Results

### Which adaptation strategies, by whom and where?

**Fig. 3:**
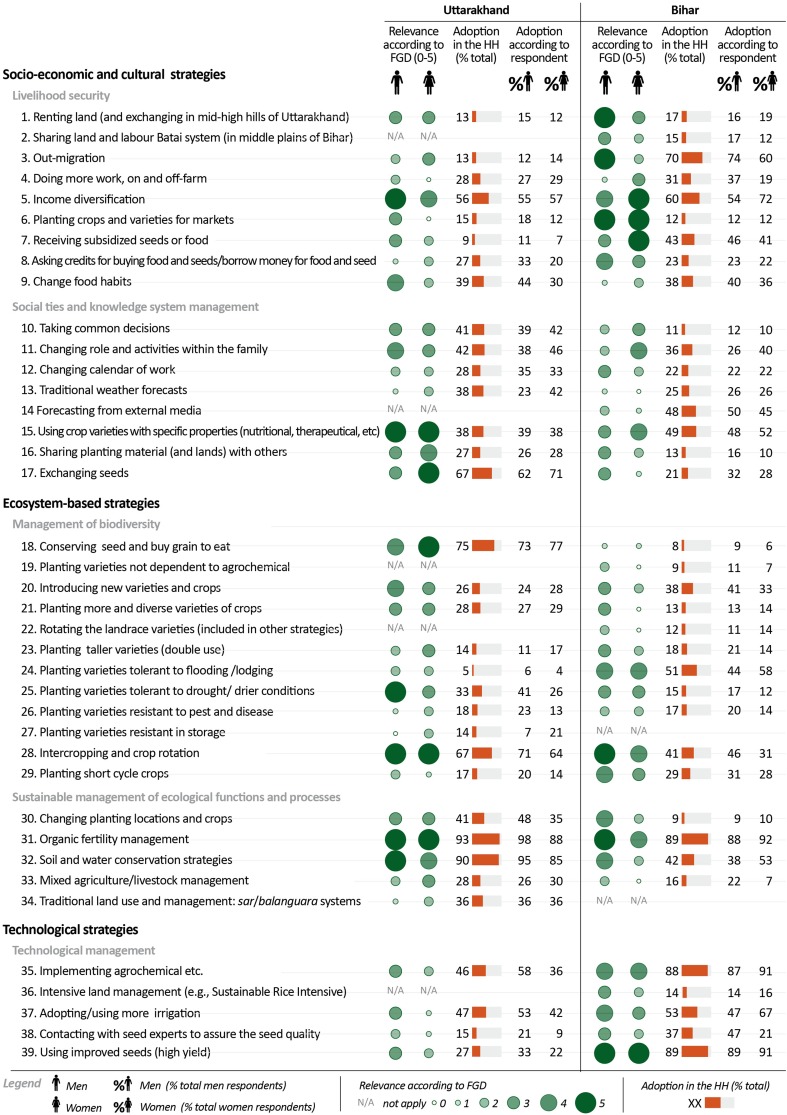
Adaptation strategies prioritized and adopted by local people in Uttarakhand and Bihar. The relative importance of adaptive strategies to multiple drivers of change is expressed by the size of circles. The degree of adoption of each of the strategies is expressed by the *orange bar* as % of respondents. Gender differences are also shown

Figure 3 shows the ranked relevance of each strategy for coping with and adapting to impacts of climate change and other concomitant drivers (first two columns, green circles), according to the prioritization made by men and women during the focus groups conducted in Uttarakhand and Bihar. It also shows the percentage of households that have adopted each strategy and the degree of adoption by respondent. A complete list of the strategies reported by the participants can be found in Supplementary material 2.

#### Socio-economic and cultural adaptation strategies in Uttarakhand

In Uttarakhand, both men and women prioritized the strategy of household income diversification, i.e. wage labour in or nearby the village, sale of home-made products, petty trade (5), as a means of guaranteeing livelihood security. Due to male out-migration (affecting 15 % of families), other strategies were also identified, such as changing the roles and tasks within the household (11) and in the calendar of work (12). Men tended to prioritize the adoption of changes in food habits (9) and the receipt of subsidized seed and food (7) as possible coping mechanisms in the case of food crisis. Interestingly, these latter options were rather questioned by women who prefer to safeguard traditional knowledge and culture of food preparation, oriented to self-sufficiency:

“I prepare traditional recipes with small quantity of black Horse gram.^4^ Men cannot sell small quantity as they do with other crops and I can save food for the next season. Therefore, I can guarantee to my children some nutritional food in the case of crisis”. (old woman, Bageshwar district)

Since women have a central role as users, conservers, knowledge holders and managers of agro-biodiversity, they highly prioritized and mainly adopt seed exchange (17):

“We are mainly farmers here. There are differences, but all women from group to group we participate in undertake collective work and we exchange seeds within the village and out of the village”^5^ (young woman, Nainital district)

To guarantee food security in the face of climate change, both men and women prioritized the adoption of traditional crop and varieties with specific nutritional properties (15), especially millets, pseudocereals (e.g. *Amaranths*) and all pulses. It is worth noting that other collective adaptive strategies were also prioritized and adopted, such as taking common decisions on land use and management (10) and sharing planting materials and land among farmers, in the case of crisis (16):

“If some year I lose my seed bank due to an extreme climate event, I can decide together with other neighbours what to do and share seed or I can move to some of their land with more favourable condition” (man, Nainital district).

Finally, the forecasting of climate events through natural elements (such as wings of ants, birds, moon, winds) was mainly adopted by women as an effective adaptation strategy (13).

#### Socio-economic and cultural adaptation strategies in Bihar

In Bihar, two socio-economic strategies are mainly prioritized and adopted by men to generate additional income. These are associated with the renting out of land (1) and out-migration (3). Although some well-off women positively perceive and adopt income diversification (5), this strategy is perceived as undesirable by poor women because it entails more seasonal work in the fields (4):

“We have a greater workload in order to ensure the survival of our families, so we have less time for other activities and for working together” (women from low caste FGD, Vaishali district)

Among other gendered differences in the perception and adoption of strategies to cope with changes, women mainly prioritized changing tasks within the family (11) and receiving subsidized seeds and food (7). Planting crops and varieties with nutritional properties for markets was also stated as important by farmers for both men and women, although this was not widely adopted as a strategy (6). In contrast, very poor and low caste household members widely adopted decreasing consumption as a mechanism of change in food habits in the case of crisis (9), which deepens the patronage relationships within the village. Finally, contrary to Uttarakhand, the forecasting of climate events using social media (e.g. TV, radio and newspaper) and mobile phones was highly adopted in Bihar (14).

#### Ecosystem-based adaptation strategies in Uttarakhand

In Uttarakhand, both men and women had a high preference for strategies of agro-biodiversity management to support sustainable ecological functions and processes. Indeed, some participants in the focus groups stated that:

“Even if we don’t have grains to eat, we don’t eat our seed, but we maintain the original material (18)” (woman, Nainital district)

Both men and women prioritized and adopted the management of specific local landraces, more suitable for responding to local environment and climatic variability. While men prioritized varieties that are able to adapt to prolonged drought and drier conditions (25), women prioritized planting taller varieties to guarantee their use for feeding both people and animals (23):

“The crops and varieties have different functions and if we lost tall varieties, we cannot feeding both children and cows”. (women, FGD, Bageshwar district)

Other agro-biodiversity strategies were also to some extent prioritized as responses to changing climate conditions, such as introducing more varieties and crops and planting more and diverse varieties and crops (20 and 21), as well as planting diverse crops and varieties together (i.e. intercropping and crop rotation) (28), which was widely adopted to guarantee minimizing the risk of losses. Men and women highlighted that such strategies facilitate the flow of new genetic material and the renewal of the diversity in fields:

“Climate is changing, we are now reintroducing new and diverse crop species and varieties for experimenting from areas with similar biophysical conditions, even if they are long away” (men, FGD, village Moura, Nainital district)

However, in the most distant and less favourable villages, there was a shift towards permanent crops:

“We don’t know the climate, so we have changed our crops, introducing permanent crops. They also increase our income.” (men, FGD, village Kilor, Nainital district)

Among the strategies for the management of ecological functions and processes, both men and women prioritized the maintenance of soil fertility through organic amendments (31), usually carried out collectively by women, and the soil and water conservation (32), usually carried out by men. Although they are widely adopted in more remote villages of Nainital district, the traditional and complex common use and management systems (34) are only partially considered as relevant to deal with climatic crises.

#### Ecosystem-based adaptation strategies in Bihar

In Bihar, most of the agro-biodiversity strategies were more highly prioritized by men than women. The strategies highly prioritized by farmers include intercropping and crop rotation systems (28), planting short-cycle crop species (29) and planting traditional varieties of rice resistant to flooding (24). This last strategy was highly adopted by women, while men mainly adopted the introduction of new varieties and crops (20). Men also prioritized some strategies for the sustainable management of ecological functions and processes, such as changing planting location and crops (30), implementing soil and water conservation strategies (32) and management of soil organic fertility (31). While this last strategy was highly adopted by men and women, conservation of water and soil management was mostly adopted by women.

#### Technological adaptation strategies

In Uttarakhand, technological strategies were not prioritized comparing to ecosystem-based and socio-cultural strategies. Despite this, the use of agrochemical inputs (35) and irrigation schemes (37), whose access usually reflected socio-economic differences within the villages, were highly adopted particularly by men. Farmers stated that they also lost landraces and started introducing high-yield improved varieties (39) as short-term response to climatic crisis and shortages of labour. However, they recognized:

“New varieties are not suitable to this climate, environment and insect attack, for this reason we are now with problems” and “There are not external experts who may help us with seeds, only old people and especially women are our experts.” (men, FGD, Bageshwar district)

In contrast, in Bihar, farmers (men and women) highly prioritized and adopted technological initiatives, such as the use of agrochemicals (35), adoption of irrigation (37) and the use of improved seeds (39). With regard to the latter strategy, farmers aimed to assure the quality of seeds through fostering training by experts and scientists:

“In the case of food and seed crises we can only rely on external aid interventions, while to respond to long-term change we prioritized planting high yielding and marketable species and varieties”. (women, FGD, Muzaffarpur district)

### Intersectional factors determining adaptation strategies in Uttarakhand

**Fig. 4:**
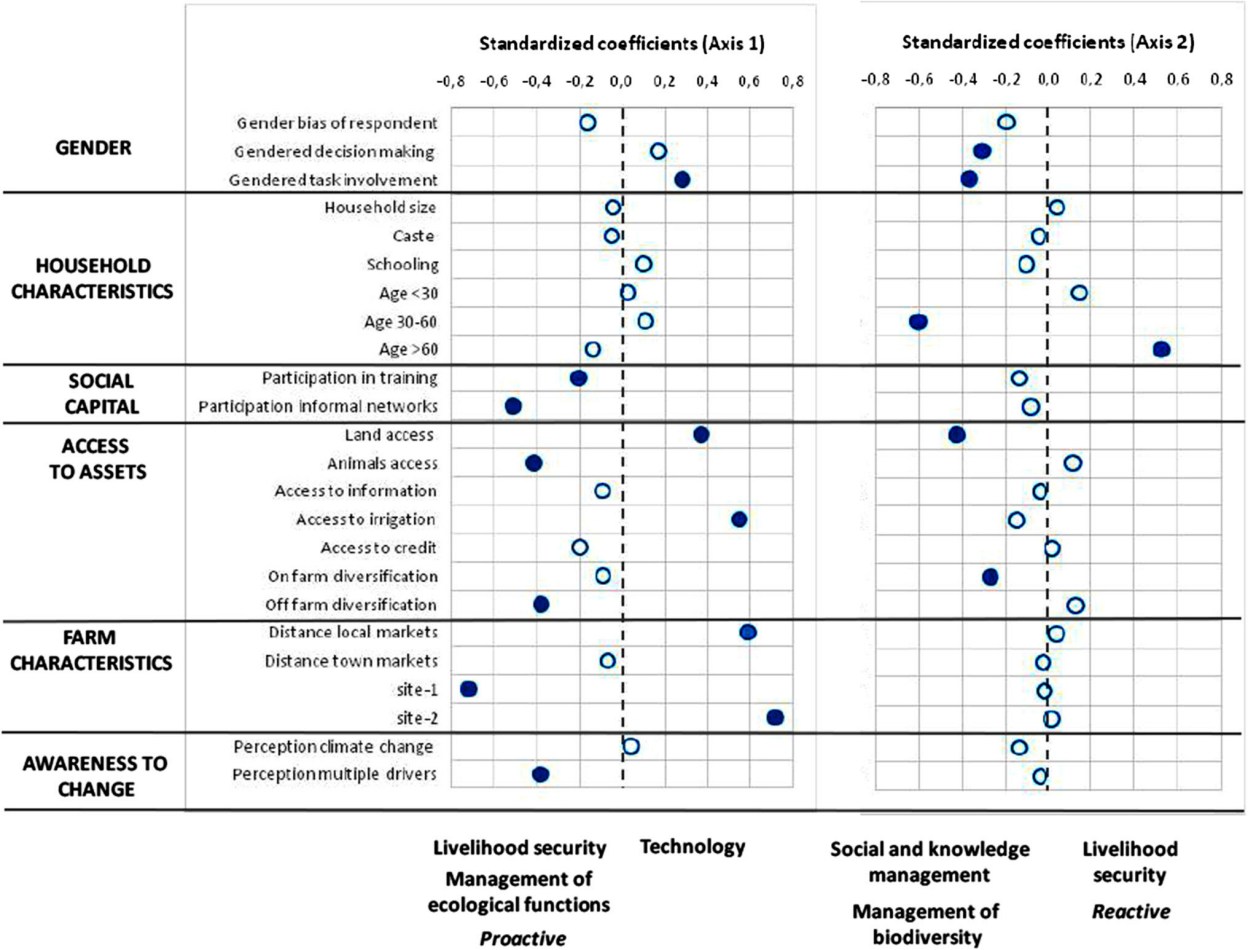
Graphical visualization of the standardized coefficients that shows the effect of each explanatory variable in each of the adaptive strategies implemented in Uttarakhand. The *blue dot* indicates that such an effect is statistically significant. Table 1 shows more details of the canonical correspondence analysis (CCA)

CCA was used to identify the underlying variables that influence the adoption of adaptation strategies in Uttarakhand (Monte Carlo permutation test, *p* < 0.01). The CCA revealed that two axes explained 70.7 % of the total variance of adaptation strategies: axis 1 (49.4 % of variance) showed a gradient of strategies from proactive responses (livelihood security and management of ecological functions and processes) to technological solutions, and axis 2 (21.3 % of variance) represented a gradient between the management of societal ties and knowledge as well as the management of agro-biodiversity and reactive strategies related with livelihood security (Fig. 4; Table 1).

**Table 1:** Results from the canonical correspondence analyses (CCA) applied to adaptive strategies in the mountain Kumaoni region of Uttarakhand and in the middle Indian Gangetic plain of Bihar. Eigenvalues, cumulative percentage variance and factor scores of the adaptive strategies in each research zone are presented. The significant contribution of each strategy to each axis is highlighted in bold

	Uttarakhand		Bihar	
	Axis 1	Axis 2	Axis 1	Axis 2
Eigenvalue	0.14	0.06	0.06	0.03
Variance explained (%)	49.42	21.25	49.04	28.22
Cumulative % of variance	49.42	70.67	49.04	77.25
Adaptive strategies				
Reactive strategies	0.009	**0.126**	**0.098**	**0.036**
Proactive strategies	**−0.040**	−0.036	−0.006	0.022
Management of biodiversity	0.021	**−0.091**	**−0.088**	**−0.046**
Management of ecological functions	**−0.078**	0.002	**−0.138**	**0.077**
Livelihood security	**−0.119**	**0.093**	**0.080**	0.011
Societal ties and knowledge management	0.001	**−0.093**	−0.021	**−0.136**
Technology	**0.342**	**0.038**	**0.044**	0.010

For axis 1, variables that explained proactive strategies related to the management of ecological functions and processes and livelihoods were (i) site 1 (Nainital district) where agriculture and livestock management are highly constrained by slope, altitude and isolation from markets, (ii) variables related with social capital, such as participation in training activities and informal networks, (iii) access to diverse off-farm activities and to animals and (iv) the awareness of multiple drivers of change besides climate change (blue dots in Fig. 4). By contrast, strategies based on technology were mainly influenced by (i) households where women have a key role in agricultural tasks, (ii) access to land and irrigation, (iii) longer distances between local markets and the farms, as well as by (iv) being located in site 2 (Bageshwar district), which is characterized by gentle hills and lowlands (blue dots in Fig. 4).

For axis 2, the management of societal ties and knowledge and the management of agro-biodiversity were explained by (i) gender-sensitive variables, (ii) age and (iii) access to land and on-farm diversification. Here, households where middle-aged women have a major involvement in agricultural tasks and decision-making were more likely to develop strategies to manage societal ties and knowledge, as well as agro-biodiversity (blue dots in Fig. 4). Finally, reactive strategies of livelihood security were mostly adopted by farmers older than 60 years of age.

### Intersectional factors determining the adaptation strategies in Bihar

**Fig. 5:**
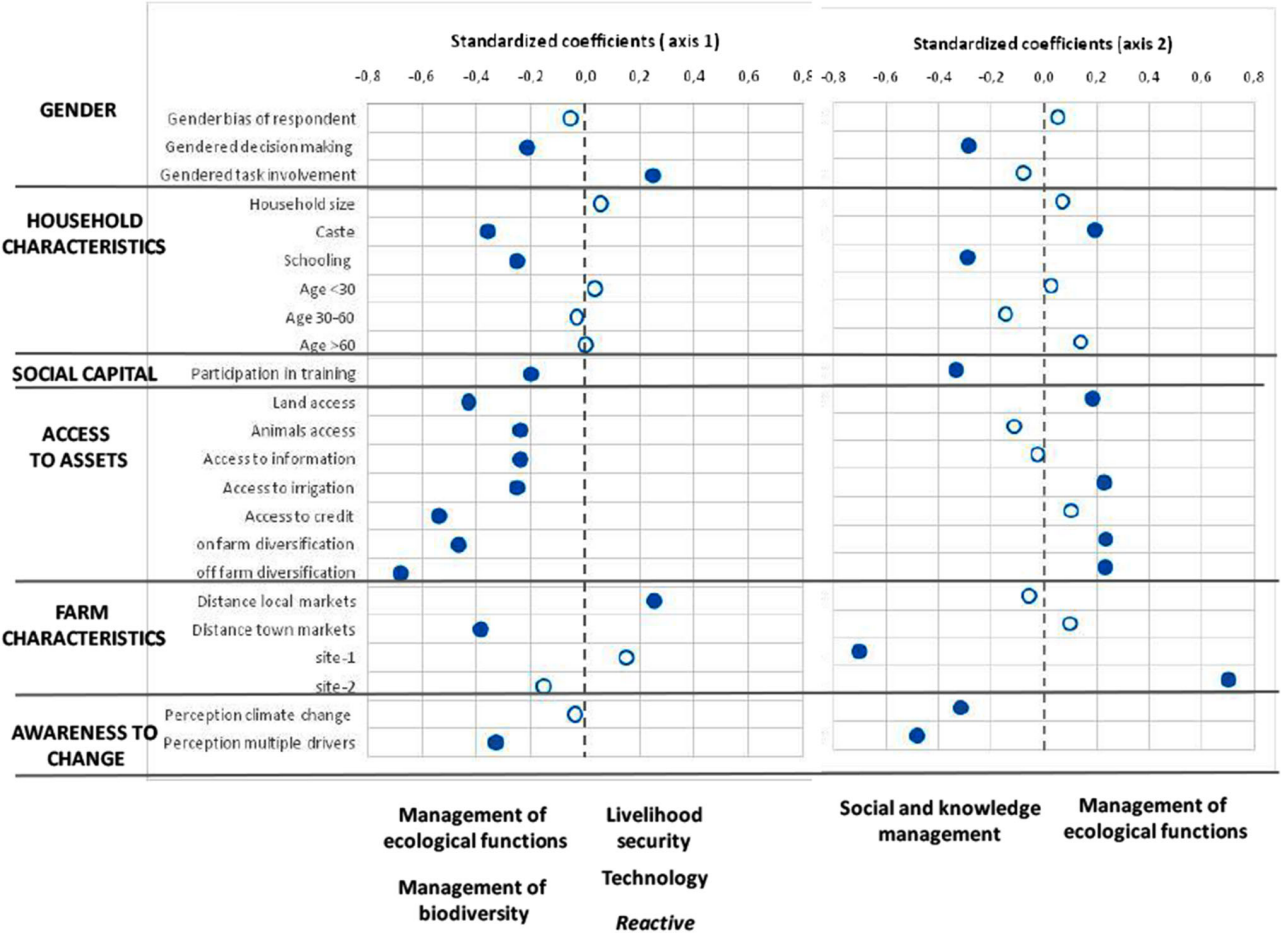
Graphical visualization of the standardized coefficients that shows the effect of each explanatory variable in each of the adaptive strategies implemented in Bihar. The *blue dot* indicates that such an effect is statistically significant. Table 1 shows more details of the canonical correspondence analysis (CCA)

According to the CCA, the first two axes captured 77.3 % of the total variance of adaptation strategies adopted by the farming communities in Bihar (Monte Carlo permutation test, *p* < 0.01; Table 1). While axis 1 (49.0 % of the variance) represented a gradient between reactive strategies, related to livelihood security and technology, and ecosystem-based adaptation strategies, axis 2 (28.2 % of the variance) distinguished between the management of ecological functions and processes and the management of agro-biodiversity as well as societal ties and knowledge (Fig. 5; Table 1).

Ecosystem-based adaptation strategies were mostly explained in axis 1 by (i) the involvement of women in decision-making, (ii) households with higher education levels and belonging to higher castes, (iii) access to multiple assets such as land, information, irrigation, credit, number of animals owned and diversification of on-farm and off-farm activities, (iv) longer distances between the farm and town markets and (v) perception of multiple drivers of change (blue dots in Fig. 5). Reactive strategies related with livelihood security were explained by (i) the involvement of women in farming tasks and (ii) by longer distances between the farm and local markets (blue dots in Fig. 5).

In particularly, the management of ecological functions and processes was also explained in axis 2 by (i) the allocation of the farm in site 2 (Muzaffarpur district, flood-prone site close to research centres with a long history of external intervention on agricultural management), (ii) higher rates of access to land and irrigation, as well as diversification of on-farm and off-farm activities and (iii) by households with higher caste (blue dots in Fig. 5). By contrast, strategies based on the management of agro-biodiversity as well as social and knowledge management was explained by (i) the involvement of women in decision-making, (ii) having higher levels of education, (iii) participation in external training, (iv) being located in site 1 (Vaishali district, i.e. a drought-prone site where new groups of men and women have been organized and trained by CCAFS interventions during the last 5 years) and (v) higher levels of awareness of the impact of climate change and other drivers of change (blue dots in Fig. 5).

## Discussion

At a first tier of analysis, our findings highlight how bundles of strategies are prioritized and adopted depending on the socio-ecological context, but mediated by a gendered nature of perceptions and decision-making within the household. In the more remote areas of Uttarakhand, a sustainable management of ecological functions and processes and agro-biodiversity are prioritized as a question of livelihood security. These findings are similar to those found in other mountain contexts in the world, for example in the Himalayan mountains (Regmi et al. 2009), in the Pamir mountains (Partoev 2012; Zimmerer 2014) and in the Andean mountains (Narloch et al. 2012). As noted elsewhere, such strategies are risk-smoothing in the context of climatic variability (Kotschi 2007; Lipper and Cooper 2009; Pascual et al. 2015), especially in low-intensity agro-ecosystems (Pascual et al. 2011; Hellin et al. 2014). Women are especially concerned with complementing agro-biodiversity management with social ties and knowledge system management mainly through exchanges of knowledge and planting materials (crops and varieties in different fields). This is reflected by a major adoption of such strategies when women are making decisions within the family. However, the data show several shifts in the strategies related to agro-biodiversity and ecological functions management. These include changes towards permanent crops and the introduction of high-yield improved seeds. Such changes are more evident in the mid-plains of Bihar, where there is a long history of external interventions and technological strategies which complement the range of strategies adopted to ensure livelihood security. This result suggests that ecosystem-based strategies are not always preferred among small-holders farmers, possibly due to the trade-offs between the benefits of adaptation in the long term and the possible transition costs in the short term, such as those related to labour intensity (as suggested by Bhattarai et al. 2015). Additionally, our findings suggest that there are differences in the awareness of individual family members to change and thus different responses are preferred and adopted to cope with shock events. Hence, a priori assumptions on the basis of a male/female dichotomy are unable to lead to a comprehensive understanding of farmers’ choices, vulnerability, adaptation processes and barriers to adoption.

Therefore, at a second tier of analysis we attempt to disentangle how multiple and fragmented dimensions of identity (including gender) intersect, revealing systems of power and cross-cutting institutions, to shape situation-specific interactions between farmers and ecosystems in the context of climate change. By helping represent complex realities, such feminist intersectional approach can potentially improve our understanding on how gender relationships are crosscut by ethnicity, caste, age, wealth class and capabilities, but also shaped by factors such as knowledge, access to communication networks, risk perception and awareness and social mobilization, ultimately influencing the ability to undertake adaptive measures. Additionally, the approach demonstrates its usefulness to visualize how categories are changing and renegotiated under new drivers. Specific examples emerge from our study. In Uttarakhand, as a result of the phenomenon of economically driven male out-migration, middle-aged women with good access to land and diversified incomes (which together may be used as a proxy for measuring wealth) are not only more involved in agriculture and marketing, but are also playing a greater role in decision-making in their domestic spheres (Fig. 4, axis 2). These women have demonstrated common adaptive responses to the main drivers of change, mostly through traditional management of agro-biodiversity, networks and knowledge. Our results thus firstly confirm that there are women who especially use, conserve and manage agro-biodiversity in mountain contexts (as per Padmanabhan 2011). Secondly, we recognize that such adaptation strategies promote a female collective agency, i.e. a capacity of groups of women to make choices in order to meet their cultural and biological needs under change. These findings are consistent with other findings elsewhere (Ray-Bennett 2009; Gabrielsson and Ramasar 2013). Indeed, previous explorations in the same area (unpublished data) noted that where women have control over decision-making and agricultural tasks, they tend to cultivate more diversified fields and home gardens, capitalize on the pooling of natural and human resources and join forces in groups of collective action. This is realized including by sharing tasks in agriculture and promoting extensive networks of seed exchange that guarantee the flow of genetic material from trusted sources (as per Bellon et al. 2011 in Mexico), as well as through experimentation, conservation of genetic material and the maintenance of “safety nets”. Mostly, local varieties are conserved and exchanged, which are adapted to the local agro-ecological conditions, and have coevolved through a long process of human selection based on farmers’ preferences and traditional knowledge. Such increased ability to benefit from the unique characteristics of a range of landraces reinforces the individual and collective adaptive capacity. Nevertheless, despite these general findings on the role of women in agro-biodiversity conservation for adaptation, our quantitative analysis also indicates that gender intersects with wealth indicators in jointly shaping the decisions on how to deal with climate change (Badola et al. 2014). Therefore, as suggested by Bhattarai et al. (2015), it is not always the case that significant ecological resilience achieved through the management of ecological functions and agro-biodiversity management may be associated with gender equity. In fact, we observe that the traditional socio-economic unequal access to land and assets is still a barrier for other groups of women in the adoption of such a range of proactive strategies, increasing the vulnerability of the majority of households who face perturbations and crisis. Finally, we also highlight that age plays a role in the capacity to adopt such complementary strategies, with old men and women mainly adopting a limited number of reactive strategies related to livelihood security-based adaptation. However, similar weaknesses in our study are also evident. Further research should focus on additional factors associated with the hidden dynamics of power within groups, such as power dynamics between tenants and the landless, those related to the gendered nature of age classes or differences between migrant and non-migrant households within wealth classes. Additionally, the results leave unclear how preferences for coping mechanisms or adaptation strategies would be negotiated by younger generations in terms of their degree of participation in decision-making within the household, especially for those families where men are more likely to have migrated and roles and activities are redefined (Lambrou and Nelson 2010). Our data also suggested that gender and wealth may also depend on the context-specific biophysical and socio-economic conditions that may catalyse or constrain decisions on adaptation pathways (Fig. 4, axis 1). For instance, in the specific socio-ecological context of Bageshwar (site 2), much more connected to markets and in general to external interventions, women with more access to land and irrigation and a greater involvement in agricultural tasks favour the adoption of technological adaptation strategies. In contrast, under more marginal biophysical and socio-economic conditions, such as in Nainital (site 1), participation in training and informal networks, access to animals and diversified income, as well as awareness of multiple drivers of change, but not gender, mainly influence the priorities on management of ecological functions and proactive livelihood strategies.

As suggested by Chant (2008), stereotypes around the relationships between gender, as a homogeneous group, and the environment mask inequalities and obscure complex environmental and gender challenges. Some other interesting findings from our empirical work reinforce such an argument and evidence the dynamics of constant re-negotiation of women’s roles and responsibilities. For instance, in Bihar, our results show that a small group of women who belong to higher castes and have higher levels of education and wealth (i.e. good access to access, information, credit and animals and multiple sources of on-farm and off-farm income) are more able to re-negotiate their roles in decision-making and develop a range of proactive ecosystem-based management strategies, decreasing their vulnerability to crisis. Further, the results suggest that the participation in training and extension programmes promoted by NGOs and intergovernmental organizations and programmes has a positive influence on such adaptation process (Fig. 5, axis 1). However, this result appears to be location sensitive. For instance, higher caste women from the other site (site 2, Muzzafarpur) are neither involved in such training nor in decision-making, probably as a result of more rigid system values leading to the greater seclusion of women from public activity (including outdoor work) (Fig. 5, axis 2). By contrast, lower caste, less-educated women are less subject to such seclusion and are therefore more likely to work as agricultural labourers. These women consequently adopt mainly reactive livelihood security strategies, but, to some extent, also technological adaptation strategies, especially those related to the introduction of hybrid seeds (Fig. 5, axis 1). Our findings show lower adoption rates of ecosystem-based strategies by such group of poor women, who are net labour sellers in the area, because they are more likely than rich women, who are net labour buyers, and men to face time constraints, as a response to the increased burden of day-to-day activities as workers. This may affect their ability to participate in accessing information and community-based climate adaptation initiatives (Behrman et al. 2014) such as the ‘climate smart agriculture’ promoted by CCAFS which requires high labour inputs in the first years. As a consequence, they show a higher exposure to perturbations and crisis. In the other site (site 1, Vaishali), the access to schooling, the participation in training and the awareness of climate change are driven by external interventions and influence mainly the adoption of agro-biodiversity management and social ties and knowledge management as relevant strategies (Fig. 5, axis 2). As suggested by Mehar et al. (2016) in a recent study in the same area, our findings confirm that the awareness on a wide range of ecosystem-based strategies through extension programmes may influence their adoption. Therefore, in line with the findings of Agarwal (2000, 2009), we argue that is important to ensure the institutional integration of gender and power analysis in planning ecosystem based adaptation processes. Such integration may avoid recommendations that lead to adaptation pathways for some groups which carry potential maladaptation for others (Barnett and O’Neill 2010), thereby burdening the most vulnerable groups.

In summary, the study suggests a fragmented and complex interplay of existing and unequal gendered context-specific multiple identities and socio-economic structural differences that might shape adaptation strategies (cp. Ray-Bennett 2009; Onta and Resurreccion 2011). Access by both women and men to training and official programmes may promote divergent but complementary bundles of opportunities that households are using for adaptation. Whether this ultimately translates into the most vulnerable women contributing more to decision-making will, however, depend on how local, national and international initiatives facilitate empowerment and policy enhancement (Djoudi and Brockhaus 2011).

## Conclusion

This paper contributes to facilitating the recognition of diverse and multiple adaptation responses and the link with gender in the context of climate change. Firstly, it demonstrates that, despite the evidence of the growing impact of climate and other socio-economic drivers, there is little recognition of geographically determined and gender-sensitive preferences and adoption of options related to ecosystem-based management, livelihoods security and technology development. Secondly, the paper offers interesting insights into the complex intersection of multiple factors which differently influence farmers’ choices on the range of adaptation options and clarifies how roles, responsibilities and power dynamics are renegotiated within the household and the community, (un)empowering women. Indeed, the empirical lessons from the more remote mountain region show the emergence of a collective agency of women to decrease common vulnerabilities to climatic variability and shocks through ecosystem-based and especially agro-biodiversity-based strategies associated with knowledge and ties management. However, the household’s capabilities (i.e. access to land, assets and diversified income), but also the age of farmers are the main barriers to the adoption of such a diversity of proactive strategies. In contrast, in the middle plains of the Indian Gangetic region a complex interaction of gender and ability to accede to training and education, depending on the specific context, the caste and the rigidity of gender norms of seclusion, mainly influences the major adoption of a mixed range of agro-biodiversity-based and technological strategies. Our results thus make clear that adaptation is not a homogenous process agreed upon by all parties and there are risks of reproducing gender bias and inequalities in development policies and interventions if it is not carefully addressed. Acknowledging the possibility of maladaptation or divergent adaptation must be a first step for researchers, policy makers and civil society to facilitate, support and fund mechanisms for adaptation at local level.

Overall, this paper also contributes to recent methodological discussions about how to conceive and implement research and development projects related to climate change adaptation under a gender transformative focus. Our work suggests how to implement a feminist intersectional approach through a two-tier interdisciplinary research approach which integrates qualitative and quantitative methods and tools. Such approach and methods should be further explored, particularly as we consider that future research could benefit from more emphasis on a nuanced analysis of the intra-gender differences that shape adaptive capacity to climate change.

## Electronic supplementary material

Below is the link to the electronic supplementary material.

Supplementary material (PDF 795 kb)

## Footnotes

1 The Government of India has categorized the so-called lower castes of India under three main categories. According to the 2011 Census of India, in Uttarakhand, Scheduled Castes constitute 18 % of population, while Scheduled Tribes and Other Backward Classes constitute 3 % of population. Forward castes (middle and high castes) constitute more than 50 % of the population in this region (Source: Census of India 2011).

2 In Bihar, Scheduled Castes constitute 15 % of population, Scheduled Tribes 1.3 % of population and Other Backward Classes 51 % of population. An Extremely Backward Class also exists and is a unique categorization to the state of Bihar. Forward classes (middle and upper castes) constitute 15 % of population in Bihar (Source: Census of India 2011).

3 Indian States are administratively composed of districts. The smallest administrative subdivision of districts is known as blocks, i.e. group of villages, which only exist in rural areas.

4 The US National Academy of Sciences has identified Horse gram (*Macrotyloma uniflorum*) as a potential food source for the future, thanks to its exceptional nutrition profile, drought resistance and general hardiness (Bhardwaj and Yadav 2015).

5 Results from our survey confirm that around a 70 % of sampled women in each village declared to be involved in collective activities of fertility management, harvesting and fodder cutting, while men are little or not involved.

## References

[CR1] Acquah-de Graft H, Onumah E (2011). Farmers’ perceptions and adaptations to climate change: An estimation of willingness to pay. AGRIS On-line Papers in Economics and Informatics.

[CR2] Adger WN, Kelly PM (1999). Social vulnerability to climate change and the architecture of entitlements. Mitigation and Adaptation Strategies for Global Change.

[CR3] Agarwal B (2000). Conceptualizing environmental collective action: Why gender matters. Cambridge Journal of Economics.

[CR4] Agarwal, B. 2002. Bargaining, gender equality and legal change: The case of India’s Inheritance Laws. Discussion Paper No. 165, October. Institute of Development Studies (Sussex).

[CR5] Agarwal B (2009). Gender and forest conservation: The impact of women’s participation in community forest governance. Ecological Economics.

[CR6] Ahmed S, Fajber E (2009). Engendering adaptation to climate variability in Gujarat India. Gender and development.

[CR7] Apata, T.G., K.D. Samuel, and A.O. Adeola. 2009. Analysis of climate change perceptions and adaptation among arable food crop farmers in South Western Nigeria. *Contributed paper presented at 23rd conference of International Association of Agricultural Economists*, Beijing, China, 16–22 August 2009.

[CR8] Arora-Jonsson S (2011). Virtue and vulnerability: Discourses on women, gender and climate change. Global Environmental Change.

[CR9] Badola R, Hussain SA (2003). Conflict in paradise: Women and protected areas in the Indian Himalaya. Mountain Research and Development.

[CR10] Badola R, Ogra MV, Barthwal SC, Oberhauser A, Johnston-Anumonwo I (2014). Ecodevelopment, gender, and empowerment: Perspectives from India’s protected area communities. Gender, development and transnational feminism: Engaging feminism and development.

[CR11] Barnett J, O’Neill SJ (2010). Maladaptation. Global Environmental Change.

[CR12] Behrman, J.A., E. Bryan, and A. Goh. 2014. Gender, climate change, and group based approaches to adaptation. In *Enhancing women’s assets to manage risk under climate change: Potential for group*-*based approaches,* ed. C. Ringler, A.R., Quisumbing, E. Bryan, R. Meinzen-Dick, 3–8. Climate Change, Collective Action and Women’s Assets. Washington, DC: International Food Policy Research Institute.

[CR13] Bellon MR, Hodson D, Hellin J (2011). Assessing the vulnerability of traditional maize seed systems in Mexico to climate change. Proceedings of the National Academy of Sciences of United States of America.

[CR14] Below T, Mutabazi K, Kirschke D, Franke C, Sieber S, Siebert R, Tscherning K (2012). Can farmers’ adaptation to climate change be explained by socio-economic household-level variables?. Global Environmental Change.

[CR15] Bhardwaj J, Yadav SK (2015). Drought stress tolerant Horse gram for sustainable agriculture. In Sustainable Agriculture Review.

[CR16] Bhattarai B, Beilin R, Ford R (2015). Gender, agrobiodiversity and climate change: A study of adaptation practices in the Nepal Himalayan. World Development.

[CR17] Binswanger-Mkhizer HP, D’Souza A, Fuglie K, Wang SL, Ball E (2012). Structural transformation and agricultural productivity in India. Productivity growth in agriculture: An international perspective.

[CR18] Bisht IS, Rao KS, Bhandari DC, Nautiyal S, Maikhuri RK (2006). A suitable site for in situ (on-farm) management of plant diversity in traditional agroecosystems of western Himalaya in Uttaranchal state: A case study. Genetic Resources and Crop Evolution.

[CR19] Carr ER (2008). Between structure and agency: Livelihoods and adaptation in Ghana’s Central Region. Global Environmental Change.

[CR20] Carr ER, Thompson MC (2014). Gender and climate change adaptation in agrarian settings: Current thinking, new directions, and research frontiers. Geography Compass.

[CR21] Chant S (2008). The “feminisation of poverty” and the “feminisation” of anti-poverty programmes: Room for revision?. Journal of Development Studies.

[CR22] Crenshaw K (1991). Mapping the margins: Intersectionality, identity politics, and violence against women of color. Stanford Law Review.

[CR23] Dankelman I (2010). Gender and climate change: An introduction.

[CR24] Datta, A., and P. Rustagi. 2012. Status of women in Bihar: Exploring transformation in work and gender relations. ISS Staff Group 0. Retrieved from http://hdl.handle.net/1765/34852.

[CR25] Davis K (2008). Intersectionality as buzzword: A sociology of science perspective on what makes a feminist theory useful. Feminist Theory.

[CR26] de Wit, M. 2006. The perception of and adaptation to climate change in Africa. CEEPA Discussion Paper No. 10. CEEPA, University of Pretoria.

[CR27] Demetriades J, Esplen E (2008). The gender dimensions of poverty and climate change adaptation. IDS Bulletin.

[CR28] Deressa, T., R. Hassan, C. Ringler, T. Alemu, and M. Yesuf. 2008. Analysis of the determinants of farmers’. Discussion Papers 798. International Food Policy Research Institute, Washington DC.

[CR29] Deressa TT, Hassan RM, Ringler C (2011). Perception of and adaptation to climate change by farmers in the Nile basin of Ethiopia. The Journal of Agricultural Science.

[CR30] Djoudi H, Brockhaus M (2011). Is adaptation to climate change gender neutral? Lessons from communities dependent on livestock and forests in northern Mali. International Forestry Review.

[CR31] Food and Agriculture Organization (FAO) (2011). The state of food and agriculture 2010–2011. Women in agriculture. Closing the gender gap for development.

[CR32] Fosu-Mensah BY, Vlek PLG, MacCarthy DD (2012). Farmers’ perception and adaptation to climate change: A case study of Sekyedumase district in Ghana. Environment, Development and Sustainability.

[CR33] Füssel H-M, Klein RJT (2006). Climate change vulnerability assessments: An evolution of conceptual thinking. Climatic Change.

[CR34] Gabrielsson S, Ramasar V (2013). Widows: Agents of change in a climate of water uncertainty. Journal of Cleaner Production.

[CR35] Gandure S, Walker S, Botha JJ (2013). Farmers’ perceptions of adaptation to climate change and water in a South African rural community. Environmental Development.

[CR36] García de Jalón S, Silvestri S, Granados A, Iglesias A (2015). Behavioural barriers in response to climate change in agricultural communities: An example from Kenya. Regional Environmental Change.

[CR37] Gbetibouo GA, Hassan RM, Ringler C (2010). Modelling farmers’ adaptation strategies for climate change and variability: The case of the Limpopo Basin, South Africa. Agrekon.

[CR38] Government of Bihar. 2014. State action plan on climate change. Building resilience through development. http://admin.indiaenvironmentportal.org.in/files/file/Bihar%20Action%20Plan%20for%20Climate%20Change.pdf.

[CR39] Government of Uttarakhand. 2014. Uttarakhand action plan on climate change. Transforming crisis into opportunity. http://www.moef.gov.in/sites/default/files/Uttarakhand%20SAPCC.pdf.

[CR40] Hellin J, Bellon MR, Hearne SJ (2014). Maize landraces and adaptation to climate change in Mexico. Journal of Crop Improvement.

[CR41] Hisali E, Birungi P, Buyinza F (2011). Adaptation to climate change in Uganda: Evidence from micro level data. Global Environmental Change.

[CR42] Huynh PTA, Resurrección BP (2014). Women’s differentiated vulnerability and adaptations to climate-related agricultural water scarcity in rural Central Vietnam. Climate and Development.

[CR43] IPCC. 2013. Climate change 2013: The physical science basis. In *Contribution of WORKING Group I to the Fifth Assessment Report of the Intergovernmental Panel on Climate Change*, ed. T.F. Stocker, D. Qin, G.-K. Plattner, M. Tignor, S.K. Allen, J. Boschung, A. Nauels, Y. Xia, et al. Cambridge: Cambridge University Press.

[CR44] IPCC. 2014. *Climate change 2014*: *Impacts, adaptation, and vulnerability*. Appendix I. Glossary WG II to the 5th Assessment Report of the IPCC. Cambridge: Cambridge University Press.

[CR45] Jain A (2010). Labour migration and remittances in Uttarakhand: Case study report.

[CR46] Kaijser A, Kronsell A (2014). Climate change through the lens of intersectionality. Environmental Politics.

[CR47] Kishore, A., Joshi, P.K., and D. Pandey. 2014. Droughts, distress, and policies for drought proofing agriculture in Bihar, India. IFPRI Discussion Paper 01398. International Food Policy Research Institute (IFPRI).

[CR48] Kotschi J (2007). Agricultural biodiversity is essential for adapting to climate change. GAIA.

[CR49] Lambrou, Y., and S. Nelson. 2010. *Farmers in a changing climate. Does gender matter? Food security in Andhra Pradesh, India*. Rome: Food and Agriculture Organization of the United Nations.

[CR50] Lipper L, Cooper D, Kontoleon A, Pascual U, Smale M (2009). Managing plant genetic resources for sustainable use in food and agriculture: Balancing the benefits in the field. Agrobiodiversity conservation and economic development.

[CR51] Lykke N (2010). Feminist studies. A guide to intersectional theory, methodology and writing.

[CR52] Mall RK, Singh R, Gupta A, Srinivasan G, Rathore LS (2006). Impact of climate change on Indian agriculture: A review. Climatic Change.

[CR53] Marino E, Ribot J (2012). Adding insult to injury: Climate change and the inequities of climate intervention. Global Environmental Change.

[CR54] McCall L (2005). The complexity of intersectionality. Journal of Women, Culture and Society.

[CR55] McLaughlin P, Dietz T (2008). Structure, agency and environment: Toward an integrated perspective on vulnerability. Global Environmental Change.

[CR56] Mehar M, Mittal S, Prasad N (2016). Farmers coping strategies for climate shock: Is it differentiated by gender?. Journal of Rural Studies.

[CR57] Mehta, M. 2008. *Gender Assessment of Uttarakhand Livelihoods.* Improvements Project in the Himalayas. Dehradun: International Center for Integrated Mountain Development.

[CR58] Ministry of Home Affairs, Government of India Bihar Population Census data. 2011. Accessed November 20, 2015, from http://www.census2011.co.in/census/state/bihar.html.

[CR59] Mitchell, T., T. Tanner, and K. Lussier. 2007. *We know what we need: South Asian women speak out on climate change adaptation*. Brighton: ActionAid and Institute of Development Studies (IDS) at the University of Sussex.

[CR60] Morton JF (2007). The impact of climate change on smallholder and subsistence agriculture. Proceedings of the National Academy of Sciences of United States of America.

[CR61] Narloch U, Pascual U, Drucker A (2012). Collective action dynamics under external rewards: Experimental insights from Andean farming communities. World Development.

[CR62] Nautiyal A (2003). Women and development in the Garhwal Himalayas. Asian Journal of Women Studies.

[CR63] Nautiyal S, Bisht V, Rao KS, Maikhu RK (2008). The role of cultural values in agrobiodiversity conservation: A case study from Uttarakhand, Himalaya. Human Ecology.

[CR64] Nellemann C, Verma R, Hislop L (2011). Women at the frontline of climate change: Gender risks and hopes. A rapid response Assessment.

[CR65] Nhemachena C, Hassan R (2008). Determinants of climate adaptation strategies of African farmers: Multinomial choice analysis. African Journal of Agricultural and resource Economics.

[CR66] Nielsen JØ, Reenberg A (2010). Cultural barriers to climate change adaptation: A case study from Northern Burkina Faso. Global Environmental Change.

[CR67] Nightingale AJ (2011). Bounding difference: Intersectionality and the material production of gender, caste, class and environment in Nepal. Geoforum.

[CR68] O’Brien KL, Leichenko R, Kelkarc U, Venemad H, Aandahl G, Tompkins H, Javed A, Bhadwal S (2004). Mapping vulnerability to multiple stressors: Climate change and globalization in India. Global Environmental Change.

[CR69] Ogra MV (2008). Human–wildlife conflict and gender in protected area borderlands: A case study of costs, perceptions, and vulnerabilities from Uttarakhand (Uttaranchal), India. Geoforum.

[CR70] Ogra MV, Badola R (2015). Gender and climate change in the Indian Himalayas: Global threats, local vulnerabilities, and livelihood diversification at the Nanda Devi Biosphere Reserve. Earth System Dynamics.

[CR71] Ojea E (2015). Challenges for mainstreaming ecosystem-based adaptation into the international climate agenda. Current Opinion in Environmental Sustainability.

[CR72] Onta N, Resurreccion BP (2011). The role of gender and caste in climate adaptation strategies in Nepal. Mountain Research and Development.

[CR73] Opiyo F, Wasonga OV, Nyangito MM, Mureithi SM, Obando J, Munang R (2015). Determinants of perceptions of climate change and adaptation among Turkana pastoralists in northwestern Kenya. Climate and Development.

[CR74] Padmanabhan M (2011). Women and men as conservers, users and managers. A feminist social–ecological approach. Journal of Socio-Economics.

[CR75] Partoev K (2012). Preservation of agrobiodiversity and community adaptation to climate change in mountain of Tajikistan. Oecologia Montana: International Journal of Mountain Ecology.

[CR76] Pascual U, Narloch U, Nordhagen S, Drucker AG (2011). The economics of agrobiodiversity conservation for food security under climate change. Economía Agraria y Recursos Naturales.

[CR77] Pascual U, Termansen M, Hedlund K, Brussaard L, Faber J, Foudi S, Lemanceau P, Liv-Jørgensen S (2015). On the value of soil biodiversity and ecosystem services. Ecosystem Services.

[CR78] Pokhriyal HC (1994). Human environment and socio-economic development in the Himalayas: An institutional study of natural and human resource management in the Garhwal and Kumaon Himalayas.

[CR79] Rao EK (2006). Role of women in agriculture: A micro level study. Journal of Global Economy.

[CR80] Ravera, F., D. Tarrasón, and E. Simelton. 2011. Envisioning adaptive strategies to change: Participatory scenarios for agropastoral semiarid systems in Nicaragua. *Ecology and Society* 16: 20. Retrieved from http://www.ecologyandsociety.org/vol16/iss1/art20/.

[CR81] Ray-Bennett NS (2009). The influence of caste, class and gender in surviving multiple disasters: A case study from Orissa, India. Environmental Hazards.

[CR82] Regmi BR, Thapa L, Suwal R, Sharma GB, Khadka S, Tamang BB (2009). Role of agrobiodiversity in promoting community based adaptation in Nepal. Journal of Forest and Livelihood.

[CR83] Resurreccion, B. 2011. The gender and climate debate: More of the same or new pathways of thinking and doing? Asia Security Initiative Policy Series No. 10. Singapore: RSIS Centre for Non-Traditional Security (NTS) Studies.

[CR84] Rodenberg, B. 2009. Climate change adaptation from a gender perspective. A crosscutting analysis of development-policy instruments. Discussion Paper 24/2009. Bonn: Deutsches Institut für Entwicklungspolitik.

[CR85] Sehgal VK, Singh MR, Chaudhary A, Jain N, Pathak H (2013). Vulnerability of agriculture to climate change: District level assessment in the Indo-gangetic Plains.

[CR86] Shiva V, Bhatt VK (2009). Climate change at the third pole: The impact of climate instability on Himalayan ecosystems and Himalayan communities.

[CR87] Sinha CP (2011). Climate change and its impacts on the wetlands of North Bihar. India Lakes & Reservoirs: Research & Management.

[CR88] Smit B, Wandel J (2006). Adaptation, adaptive capacity and vulnerability. Global Environmental Change.

[CR89] Smit B, Burton I, Klein R, Wandel J (2000). An anatomy of adaptation to climate change and variability. Climatic Change.

[CR90] Sofoluwe N, Tijani A, Baruwa O (2011). Farmers’ perception and adaptations to climate change in Osun State, Nigeria. African Journal of Agricultural Research.

[CR91] Sugden F, Maskey N, Clement F, Ramesh V, Philip A, Rai A (2014). Agrarian stress and climate change in the Eastern Gangetic plains: Gendered vulnerability in a stratified social formation. Global Environmental Change.

[CR92] Sultana F (2014). Gendering climate change: Geographical insights. The Professional Geographer.

[CR93] Terry G (2009). No climate justice without gender justice: An overview of the issues. Gender and Development.

[CR94] Tschakert P (2012). From impacts to embodied experiences: Tracing political ecology in climate change research. Danish Journal of Geography.

[CR95] Vignola R, Harvey CA, Bautista-Solis P, Avelino J, Rapidel B, Donatti C, Martinez R (2015). Ecosystem-based adaptation for smallholder farmers: Definitions, opportunities and constraints. Agriculture, Ecosystems & Environment.

[CR100] WEDO-IUCN. 2014. *United Nations Framework Convention on Climate Change (UNFCCC). Decisions and conclusions: Existing mandates and entry points for gender equality*. http://www.wedo.org/wp-content/uploads/GE-Publication-ENG-Interactive.pdf.

[CR96] Zimmerer, K.S. 2014. Conserving agrobiodiversity amid global change, migration, and nontraditional livelihood networks: The dynamic uses of cultural landscape knowledge. *Ecology and Society* 19: 1. Retrieved from 10.5751/ES-06316-190201.

