# Supplementary material for: The diversity of gendered adaptation strategies to climate change of Indian farmers: A feminist intersectional approach
Source: Ambio. 2016 Nov 22;45(Suppl 3):335–51. doi: 10.1007/s13280-016-0833-2 (PMC5120025; doi:10.1007/s13280-016-0833-2)
Supplement: Supplementary file 1 — Supplementary material (PDF 795 kb) [file 13280_2016_833_MOESM1_ESM.pdf]

***Ambio***

Electronic Supplementary Material

Title: **The diversity of gendered adaptation strategies to climate change of Indian farmers: A feminist intersectional approach**

Authors: Federica Ravera, Berta Martín-López, Unai Pascual, Adam Drucker

**Supplementary material 1**

List of variables selected from recent literature to be used in the Canonical Correspondence Analyses (CCA) as explanatory factors, determining the adoption of different adaptive strategies. Note: to reduce heteroscedasticity, continuous variables were transformed into *ln (x+1).*

| **Variables** | **Literature** | **Description /measure** | **UK** (N=135) | **Bihar** (N=176) |
|---|---|---|---|---|
| ***Gender variables*** | | | | |
| Gender bias of respondent | Acquah-deGraf and Onumah (2011); Below et al. 2012; Fosu-Mensah et al. (2012); Huynh and Resurreccion (2014); Opiyo et al. (2015) ; Ray-Bennett (2009); Sofoluwe et al. (2011); Deressa et al. (2008); Gbetibouo et al. (2010) | Female respondent (Dummy, takes the value of 1 if respondent is female) (% HH =1) | 51% | 41% |
| Gendered decision making | | Decision making in agriculture is controlled by women (dummy, takes the value of 1 if there is control) (% HH =1) | 42% | 30% |
| Gendered task involvement* | | Main involvement of women in tasks of agriculture (dummy, takes the value of 1 if there is involvement) (% HH =1) | 59% | 37% |
| ***Intersectional HH variables*** | | | | |
| HH size | Deressa et al. (2008, 2011); Opiyo et al. (2015); Sofoluwe et al. (2011) | Number of people in the house (nº) Mean (SD) | 6.7 (2.9) | 9.7 (4.6) |

| Variables | Literature | Description /measure | UK<br>(N=135) | Bihar<br>(N=176) |
|---|---|---|---|---|
| Schooling | Acquah-deGraf and Onumah (2011); Apata et al. (2009) ; Deressa et al. (2011) ; García de Jalón et al. (2015); Below et al. 2012; Opiyo et al. (2015); Gbetibouo et al. (2010) | Years in school completed by HH head (nº)<br>Mean (SD) | 2.9 (1.5) | 2.5 (1.4) |
| Age | Acquah-deGraf and Onumah (2011); Deressa et al. (2008); García de Jalón et al. 2015; Hisali et al (2011) ; Below et al. (2012) | Age HH head (years)<br>Mean (SD) | 50 (15.6) | 46 (14.9) |
| Caste / Social class | Onta and Resurreccion (2011); Huynh and Resurreccion (2014); Ray-Bennett (2009) | Respondent belong from different caste (ordinal from low=1 to high=3)<br>(% HH ) | Low=6%<br>Medium=79%<br>High=15% | Low=3%<br>Medium=20%<br>High=76% |
| ***Social capital*** | | | | |
| Participation in agricultural extension/training programs | Below et al. (2012); Gbetibouo et al. (2010) | Participation (Dummy, takes the value of 1 if there is participation) (% HH =1) | 46% | 38% |

| Variables | Literature | Description /measure | UK (N=135) | Bihar (N=176) |
|---|---|---|---|---|
| Participation in informal networks/institutions | Below et al. (2012) | Participation (Dummy, takes the value of 1 if there is participation) (% HH =1) | 51% | 5% |
| ***Access to assets (wealth)*** | | | | |
| Land access | Acquah-deGraf and Onumah (2011); Below et al. (2012); Fosu-Mensah et al. (2012) ; García de Jalón et al. (2015) ; Hisali et al (2011) ; Gbetibouo et al. (2010) | Cultivated land (own and rented land) (ha)<br>Mean (SD) | 0.4 ( 0.7) | 1.4 (1.9) |
| | | Land ownership categories<br>(% HH) | Landless=1,6%<br>Small farmers (<0.4 ha) =72%<br>Medium Farmers (0.4-1.2) =21.4%<br>Large farmers (>1.2 ha)=5% | Landless=6.7%<br>Small farmers (<0.5 ha) =33.5%<br>medium farmers (0.5-2) =42.8%<br>Large farmers (>2 ha) =17% |
| Animals access | Deressa et al. (2011) ; García de Jalón et al. 2015 ; Opiyo et al. (2015) ; Sofoluwe et al. (2011) | Total number of animals owned (nº)<br>Mean (SD) | 4.0 (3.1) | 1.8 (1.5) |

| Variables | Literature | Description /measure | UK (N=135) | Bihar (N=176) |
| --- | --- | --- | --- | --- |
| Access to information | de Wit (2006); García de Jalón et al. 2015 ; Hisali et al (2011) | Level of access to information (from high=4 to null=0) | 1.9 (0.6) | 1.6 (0.8) |
| Access to irrigation | de Wit (2006); Deressa et al. (2011); Gbetibouo et al. (2010) | Level of access to irrigation (from high=4 to null=0) | 0.4 (0.7) | 1.8 (0.6) |
| Access to credit | Deressa et al. 2008; de Wit (2006); Fosu-Mensah et al. (2012); Hisali et al (2011) | Access to credit (Dummy takes the value of 1 if there is access; % HH =1) | 40% | 39 % |
| Off-farm income diversification | Acquah-deGraf and Onumah (2011); Nielsen and Reenberg (2010); Sofoluwe et al. (2011); García de Jalón et al. 2015 ; Nhemachena and Hassan (2008); | Number of off farm activities income-generating (nº) | 2.6 (1.4) | 1.8 (1.1) |
| On-farm income diversification | | Number of on-farm activities mainly oriented to market (nº) | 1.0 (1.2) | 1.9 (1.6) |
| ***Farm location*** | | | | |
| Distance to market place | Below et al. (2012); Garcıa de Jalon et al. (2014) | Distance to close (farmers') markets (Km) | 4.7 ( 4.0) | 4.0 ( 1.4) |
| | | Distance to town markets (Km) | 39.4 (3.0) | 21.0 (4.7) |
| Location (site) | Below et al. (2012); García de Jalón et al. (2015) ; Gbetibouo et al. (2010) | Agro-ecological zone (% HH in site 1 and % HH 2) | Site 1=70%<br>Site 2=30% | Site 1=56%<br>Site 2=44 % |

| Variables | Literature | Description /measure | UK (N=135) | Bihar (N=176) |
|---|---|---|---|---|
| ***Perceptions of change*** | | | | |
| Perception of climate change drivers | Below et al.(2012); Gandure et al. (2013) | Number of weather related changes (T, rainfall etc.) perceived by a household within the last decade: $Pij = \Sigma\, i(n)_j$ with:<br>*Pij = Number of perceived changes by jth household*<br>*i…m = Changes of weather parameters*<br>Mean (SD) | 4.0 (1.1) | 2.9 (2.0) |
| Perception of multiple drivers of change (including environmental change) | | Number of changes (socio-cultural, economic, political, environmental) perceived by a household within the last decade: $Pij = \Sigma\, i(n)_j$ with:<br>*Pij = Number of perceived changes by jth household*<br>*i…m = Changes of parameters*<br>Mean (SD) | 9.7 (2.5) | 8.8 (3.8) |

* We developed the following categories of gender tasks division of work: "tasks mainly managed by men" (when more than 5 tasks over 9 were managed and controlled by men of the family), "tasks mainly managed by women" (more than 5 tasks over 9 were managed and controlled by women of the family), "tasks with shared management" (when tasks were managed and controlled by both men and women). In a few number of cases where less than five tasks had been identified as managed by one category, we assigned the more cited category.

**Supplementary material 2**

Social perceptions of main drivers, impacts and strategies adopted. Note: number of strategies is defined in Figure 2.

| Drivers | Main impacts (% respondents) | Strategies adopted |
|---|---|---|
| ***Uttarakhand*** | | |
| *Climate change*<br>[increased temperature; more frequent droughts; later start of rains; uncertainty of climatic events and erratic monsoon; less overall rainfall; spring water changes; decreasing on snowfall] | Woman moves far away for NR (e.g. water, fuelwood) (82%)<br>Yield decline due to climatic stress (67%)<br>Woman works more in the field (57%)<br>Food availability decrease (47%)<br>Pest and diseases (41%)<br>Change in growing season (12%)<br>Loss of species/varieties (7%)<br>Cropping change (7%) | Socio-economic and cultural (4, 7, 8, 10, 11, 12, 13, 15, 16, 17) /<br>Ecosystem-based (18, 20, 21, 23,24,25,26,27,28,29, 30, 32, 34) /<br>Technological (35, 37, 38, 39) |
| *Land use change and environmental changes* | Soil degradation and yield decline (91%)<br>Woman moves far away for NR (e.g. water, fuelwood) (82%)<br>Loss of forest cover (76%)<br>Decreasing availability of fuel wood and other NWFP (42%)<br>Land fragmentation (35%)<br>Wildlife movement near to villages due to forest loss (6%)<br>Decreasing pasture and fodder (6%) | Socio-economic and cultural (1, 4, 6, 10, 11, 12, 15, 17) /<br>Ecosystem-based (20, 21, 23, 28, 30, 31, 32) /<br>Technological (35, 36, 37, 39) |
| *Political and economic change* | High price of food and dependence from external | Socio-economic and cultural (1, 3, 4, 5, |

| Drivers | Main impacts (% respondents) | Strategies adopted |
|---|---|---|
| | intervention (76%)<br>Woman works more in the field (57%)<br>Out-migration and less labor force availability (46%)<br>Increasing external interventions in seed and agriculture management(40%) | 6, 7, 8, 9, 10, 11, 12, 15) /<br>Ecosystem-based (18, 19, 33) /<br>Technological (36) |
| *Cultural Changes* | Changes of customs, habits etc. (71%)<br>Changing woman/man relations and expectations young people (46%)<br>Less leadership in the village (16%)<br>More willingness with environmental issues (10%) | Socio-economic and cultural (3, 9, 10, 11, 12, 15, 17) /<br>Ecosystem-based (18) /<br>Technological (-) |
| ***Bihar*** | | |
| *Climate change*<br>[increased temperature; later start of rains; uncertainty of climatic events and erratic monsoon; less overall rainfall; lower groundwater table; more frequent droughts; strong winds] | Woman works more in the field (60%)<br>Yield decline due to climatic stress (43%)<br>No changes in woman role/decision making (32%)<br>Pest and diseases (26%)<br>Change in growing season (14%)<br>Woman moves far away for NR (13%)<br>Land use and cropping change (12%)<br>Food availability decrease/food crisis (10%)<br>Loss of species/varieties (5%) | Socio-economic and cultural (1, 2, 4, 7, 8, 9, 10, 12, 14, 16, 17) /<br>Ecosystem-based (20, 21, 22, 23, 24, 25, 26, 28, 29, 30, 33) /<br>Technological (36, 37, 38, 39) |
| *Land use and environmental changes* | Increasing yield due to technological advances (71%)<br>Land fragmentation/access to cultivated land (51%)<br>Soil degradation and yield decline (14%)<br>Woman moves far away for NR (13%) | Socio-economic and cultural (1, 2, 4, 6, 12) /<br>Ecosystem-based (19, 20, 21, 22, 23, 28, 30, 31, 32, 33) /<br>Technological (35, 36, 37) |

| Drivers | Main impacts (% respondents) | Strategies adopted |
|---|---|---|
| *Political and economic change* | Out-migration and less labor force availability (92%)<br>Increasing external interventions (NGOs and governmental) in seed/agriculture management (64%)<br>Woman works more in the field (60%)<br>High price of food, inputs, irrigation and seed (and associated corruption and risk on quality of seed) (30%)<br>New policy of seeds: dependence from external sources, (20%)<br>New opportunity to sell (15%)<br>Increasing control /monitoring (11%) | Socio-economic and cultural (1, 2, 3, 4, 5, 6, 7, 8, 9, 10, 15, 16, 17) /<br>Ecosystem-based (19, 29) /<br>Technological (35, 38, 39) |
| *Cultural Changes* | Changing patterns of woman/man relations (64%)<br>Changes of customs (46%)<br>No changes in woman role/decision making (32%)<br>More willingness with environmental issues (30%)<br>Less leadership in the village (19%) | Socio-economic and cultural (3, 9, 10, 11, 12, 15) /<br>Ecosystem-based (-) /<br>Technological (-) |
